# *Vanda roxburghii*: an experimental evaluation of antinociceptive properties of a traditional epiphytic medicinal orchid in animal models

**DOI:** 10.1186/s12906-015-0833-y

**Published:** 2015-09-03

**Authors:** Md. Josim Uddin, Md. Masudur Rahman, Md. Abdullah-Al-Mamun, Golam Sadik

**Affiliations:** Department of Pharmacy, International Islamic University Chittagong, 154/A College Road, Chittagong-4203, Bangladesh; Department of Pharmacy, University of Rajshahi, Rajshahi-6205, Bangladesh

**Keywords:** Antinociceptive, Analgesic, *Vanda roxburghii*, Formalin, Cytotoxicity, Pain

## Abstract

**Background:**

Ethnopharmacological approach has explored several leads from plant sources to identify potential new drugs for various diseases including pain. *Vanda roxburghii* R. Br., an epiphytic orchid is widely distributed throughout Bangladesh. The root of this plant has a folkloric reputation to treat inflammations, fever, dyspepsia, bronchitis, hiccough, piles, snake bites, and diseases of the nervous system. In this study therefore, we aimed to investigate antinociceptive and cytotoxic properties of the extracts from the root of *V. roxburghii*.

**Methods:**

Dried powder of aerial root of *V. roxburghii* was extracted with methanol (VRM) and the resultant was fractionated into petroleum ether (VRP), chloroform (VRC), ethyl acetate (VRE), and residual aqueous fraction (VRA). The antinociceptive effect of the extracts was evaluated in mice using acetic acid-induced writhing, formalin injection, and hot plate tests. The brine shrimp lethality bioassay *in vitro* was used to evaluate cytotoxic activity of the plant extracts.

**Results:**

In the acetic acid-induced writhing test, mice treated with different fractions (12.5, 25, and 50 mg/kg, i.p.) exhibited reduced number of writhing. Amongst, VRE showed the highest activity at all three concentrations (43.65, 71.34, and 80.23 %, respectively) in a dose-dependent manner. Secondly, VRC (12.5, 25, and 50 mg/kg, i.p.) displayed the highest reduction of paw licking time in mice during the first phase of the formalin test (by 15.00, 37.05, and 56.44 %, respectively) as well as during the second phase of the test (by 20.55, 49.08, and 59.81 %, respectively). In hot plate test, VRE treatment at doses of 25 and 50 mg/kg both increased the highest latency time after 30 min. All fractions showed lower cytotoxicity compared with the standard drug vincristine sulfate in the brine shrimp bioassay.

**Conclusion:**

Overall studies suggest that the root of *V. roxburghii* is effective as a potent analgesic with lower toxicity. Our findings support previous claims of traditional uses of *V. roxburghii* in different inflammatory disorders.

## Background

Pain is an unpleasant sensory and emotional experience associated with actual or potential tissue damage. The presence of pathologic process such as tumor, muscle spasm, inflammation, nerve damage or the exposure to noxious chemical, mechanical, or thermal stimuli may be contributing factors of this damage [1]. On the other hand, chronic pain is associated with conditions such as back injury, migraine headaches, arthritis, herpes zoster, diabetic neuropathy, temporomandibular joint syndrome, and cancer [2]. Among the different pains (somatic, visceral, neuropathic, and sympathetic), generally two groups of analgesics are commonly used in the therapy of pain, steroidal and non-steroidal. Non-steroidal anti-inflammatory drugs (NSAIDs) are recommended for the therapy of mild to moderate pains, while steroidal (opioids), the oldest analgesics, are mainly used to alleviate intense acute and severe chronic pains, often post- operative and cancer-related pains. However, due to some adverse effects like gastric lesions caused by NSAIDs, and state of tolerance and dependence induced by opioids limit their free usage [3, 4]. Therefore, search of new medicinal agents which lack those adverse effects may give chance to obtain a new drugs with improved pharmacological properties, substantially helping to extend the range of therapies, effectiveness, and safety.

A number of plant-derived medicines had been used since ages without any adverse effects. Plants represent a large natural source of useful compounds that might serve as lead for the development of novel drugs [5]. It is therefore necessary efforts should be made to introduce new medicinal agents to develop more effective and cheaper drugs. In this regards, traditional medicine has been paid great attention because they are cheap, available, and have little side effects which vindicates WHO that around 80 % of the world population still rely mainly on plant-based drugs [6].

*Vanda roxhuighii* R. Br., an epiphyte belonging to the family Orchidaceae, is commonly known as Rasna widely distributed throughout Bangladesh. In all over Bangladesh, it is found on Mango, Black berry, and Guava trees. The medicinal properties of *V. roxburghii* are well described in Ayurveda, traditional Indian medicine [7]. The root of this plant has a folkloric reputation for alleviating inflammations. Traditionally, the root of *V. roxburghii* has been used to treat fever, dyspepsia, bronchitis, hiccough, piles, and snake bites [8]. In addition, externally the root is used in rheumatism and allied disorders, and diseases of the nervous system [9]. The leaves are pounded and the paste is applied to the body to bring down fever; their juice is dropped in the ear for the treatment of otitis [10]. Phytochemical investigations of *V. roxburghii* demonstrated several active compounds including heptacosane, octacosanol [11]; melianin, and 2,7,7-tri methyl bicyclo heptanes with antifungal and aphrodisiac activities, respectively [12,13]. The plant has also been reported to possess important pharmacological effects including anti-inflammatory, anti-arthritic [14], wound healing [15], anti-oxidant [16], anti-diarrheal [17], and hepatoprotective [18] effects. Previously we reported the antinociceptive activity of methanol and aqueous extracts of leaves of *V. tesselata* [19].

Although root of *V. roxburghii* has extensive medicinal use to treat pain and inflammation, to date, no studies have yet examined its antinociceptive capabilities. Therefore, the aim of this study was to evaluate the antinociceptive activity of *V. roxburghii* root in order to treatment of pain.

## Methods

### Plant material

The aerial root of *V. roxburghii* were collected from Rajshahi University campus, Rajshahi, Bangladesh, and identified by expert taxonomist of Bangladesh Forest Research Institute, Chittagong, Bangladesh. A voucher specimen (Accession No. 4306) was deposited to the herbarium of the institute.

### Extraction of plant material

The collected root was air dried and powdered. Dried powdered root (500 g) of *V. roxburghii* was extracted exhaustively with methanol by cold extraction method. The extract was then filtered and concentrated under vacuum using rotary evaporator at 50 °C temperature to obtain the crude methanol extract (12.5 g). An aliquot (10 g) of the concentrated methanol extract was dissolved in water and further fractionated into four different extractives [20]. The resultant partitionates that is petroleum ether (2.05 g), chloroform (3.68 g), ethyl acetate (1.67 g), and aqueous (2.6 g) extracts were obtained for the experiment.

### Animals

Adult Swiss albino mice (both sex) weighing approximately 30-35 g were used for this experiment. The mice were purchased from the animal research branch of the International Centre for Diarrhoeal Disease and Research, Bangladesh (ICDDR, B). The animals were maintained standard laboratory conditions (25 °C and light/dark cycles i.e. 12/12 h) and provided with standard laboratory food and distilled water ad lib. The experimental protocol was approved by the P&D committee, Department of Pharmacy, International Islamic University Chittagong, Bangladesh (Pharm-P&D-44/04’13-01).

### Drugs and chemicals

Diclofenac Na, formalin, and acetic acid were obtained from MERCK, India. Morphine sulfate and 0.9 % NaCl saline solution was obtained from Popular Pharmaceuticals Ltd., Bangladesh. Unless otherwise specified, all other reagents were of analytical grade.

### Phytochemical screening of the plant extract

Qualitative phytochemical analysis of the extracts were carried out to determine the presence of tannins, alkaloids, saponins, flavonoids, phenols, steroids, and glycosides by the methods of gelatin test, Mayer’s test, froth test, lead acetate test, ferric chloride test, Liebermann burchard’s test and legal’s test respectively as described [20].

### *In vivo* experiments: antinociceptive activity

Evidence of antinociceptive properties of root of *V. roxburghii* was investigated in the three different mice models.

#### Acetic acid-induced writhing test

Mice of either sex (n = 5) weighing 30-35 g were used and divided into 17 groups. Group I was injected with normal saline (10 ml/kg) as control, Group II received standard drug diclofenac sodium (10 mg/kg) while the remaining groups were injected with 12.5, 25, and 50 mg/kg i.p. of five fractions each. After 30 min of saline, diclofenac sodium, and plant extracts injection, the animals were treated i.p. with 1 % (v/v) acetic acid. The number of abdominal constrictions (writhes) was counted after 5 min of acetic acid injection for the period of 10 min and compared to the response in the control group [21]. Antinociceptive activity was calculated as the percentage inhibition of writhing.

#### Formalin test

The method used was similar to that described by Hunskaar and Hole, 1987 [22]. Twenty micro liters of 2.5 % formalin, made up in 0.9 % saline water, was injected into the sub-plantar area of the right hind paw of mice. Animals were pretreated i.p. with vehicle (0.1 ml/kg saline water), morphine sulfate and diclofenac sodium (10 mg/kg), and different doses of fractions (12.5, 25, and 50 mg/kg) 60 min before formalin injection. The time (in seconds) spent licking and biting the injected paw was measured as an indicator of pain response. Responses were measured for 5 min (first phase) and 15-30 min (second phase) after formalin injection corresponding to the neurogenic and inflammatory pain responses, respectively. Antinociceptive activity was calculated as the percentage inhibition of licking time.

#### Hot plate test

The hot plate test was used to measure central analgesic activity by the method described previously [23] with minor modifications. Only female mice were divided into twelve groups of six mice each. Mice were pre-selected on the hot plate maintaining a constant temperature of 55 ± 0.1 °C. Licks or flicks on the rear paws were the parameters of observation. Animals showing a reaction time (defined as the latency for licking or flicking the hind paw or jumping) greater than 20 s were discarded. The animals were then treated with vehicle (saline, 0.1 ml/10 g, i.p.), morphine sulfate (10 mg/kg, i.p.), and five fractions of the extract (25 and 50 mg/kg, i.p.). Latency time (in seconds) for each mouse was determined on the hot plate during a maximum period of 20 s at intervals of 30, 60, 90, and 120 min after the administration of the vehicle, extracts, and morphine.

### *In vitro* experiment: cytotoxicity assay

The cytotoxic potentiality of all the root extracts of *V. roxburghii* were performed on brine shrimp nauplii using Mayer’s method [24]. The eggs of the brine shrimps were hatched in artificial seawater (prepared by using sea salt 38 g/L and adjusted to pH 8.5 using 1 N NaOH) providing constant aeration for 48 h under the light to get shrimp larvae called nauplii. Each extracts was dissolved in seawater with DMSO (not exceed 0.01 %) separately and transferred to test tubes to obtain concentrations of 50, 100, 200, 300, and 400 μg/ml in 5 ml artificial seawater with 10 nauplii in each test tube. Standard drug vincristine sulfate was used as positive control at concentrations of 0.312, 0.625, 1.25, 2.5, and 5 μg/ml. Control test tubes were subjected to DMSO with seawater at the same concentration as in test tubes for test samples. After 24 h incubation period at 25-30 °C, the number of viable nauplii was counted using a magnifying glass. The median lethal concentration (LC_50_) values were calculated from linear correlation.

### Statistical analysis

One-way ANOVA followed by Dunnet’s test was used to interpret the data for significant differences between the test and control groups using GraphPad Prism Data Editor for Windows, Version 6.0 (GraphPad software Inc., San Diego, CA). Data represent mean ± standard error of mean (± SEM.) values. P values (<0.01 and <0.05) were considered as statistically significant.

## Results

### Phytochemical screening

The phytochemical screenings of different extracts were performed to detect the presence or absence of bioactive components; qualitative results are shown in the Table 1. The analyses revealed the presence of tannins, saponins, alkaloids, flavonoids, phenolics, steroids, and glycosides.

**Table 1:** Phytochemical screening of methanol extract of the *V. roxburghii* root and its different fractions

Phytochemical constituents	Extract and fractions				
	VRM	VRP	VRE	VRC	VRA
Tannins	+	-	-	-	+
Alkaloids	+	-	-	-	+
Saponins	+	-	-	-	+
Flavonoids	+	+	+	+	+
Phenolic compounds	+	+	+	+	+
Steroids	+	+	-	-	-
Glycosides	+	+	+	+	+

VRM *V. roxburghii* methanol extract, VRP *V. roxburghii* petroleum ether fraction, VRE *V. roxburghii* ethyl acetate fraction, VRC *V. roxburghii* chloroform fraction, VRA *V. roxburghii* aqueous fraction

### Antinociceptive activity

#### Acetic acid-induced writhing test

**Fig. 1:**
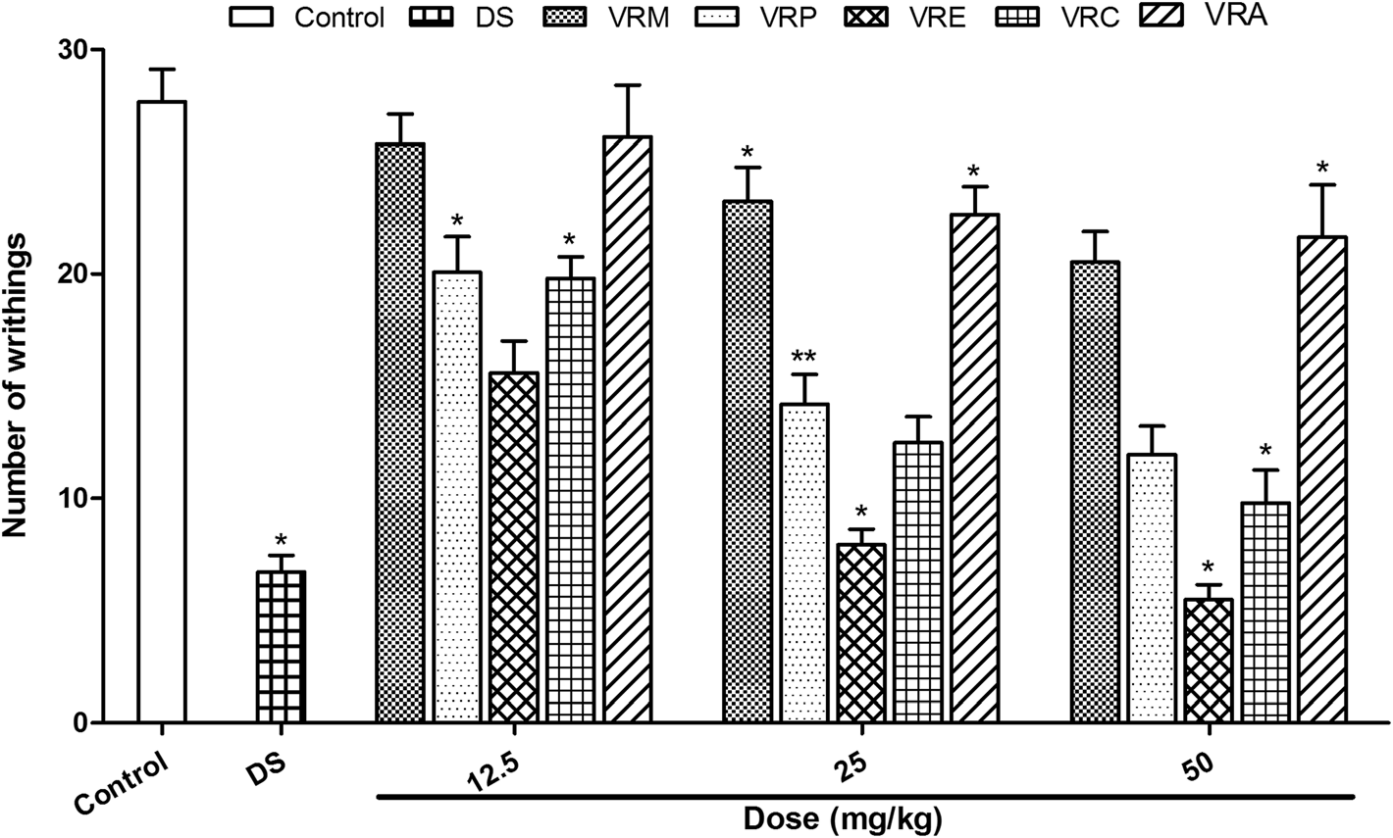
Effect of methanol extract of the *V. roxburghii* root and its different fractions, and DS (10 mg/kg) on acetic acid induced writhing test. Values are mean ± S.E.M. *p < 0.05 and **p < 0.01, significantly different from control; ANOVA followed Dunnett’s test (n = 5, per group). VRM: *V. roxburghii* methanol extract; VRP: *V. roxburghii* petroleum ether fraction; VRE: *V. roxburghii* ethyl acetate fraction; VRC: *V. roxburghii* chloroform fraction; VRA: *V. roxburghii* aqueous fraction; DS: diclofenac sodium

Reduction of pain sensation triggered by acetic acid induced writhing response is one of the remarkable procedures to evaluate the peripherally acting analgesics. The inhibition percentage of writhing movements of methanol extract derived from *V. roxburghii* (VRM) against Swiss albino mice is shown in Fig. 1. The intraperitonial administration of the extract (12.5, 25, and 50 mg/kg) showed a concentration-dependent antinociceptive effect. The VRM was found to decrease writhing by 6.80, 15.97, and 25.81 % at a concentration of 12.5, 25, and 50 mg/kg, respectively, while diclofenac sodium (10 mg/kg), used as reference standard caused 75.74 % reduction of writhing movement. To evaluate precise activity of the plant, VRM was fractionated successively with petroleum ether, chloroform, ethyl acetate , water, and the resulting fractions were tested similarly. Among the fractions, VRE showed the highest activity with 80.23 % inhibition at 50 mg/kg followed by VRC, VRP, and VRA with 64.62, 56.74, and 21.72 % inhibition, respectively under the same experimental condition. Meanwhile, the activity at lower dose 12.5 mg/kg of VRM, VRP, VRC, VRE, and VRA exhibited 6.80, 27.44, 43.63, 28.38 and 5.63 % inhibition respectively. Our results demonstrate that *V. roxburghii* has a significant peripheral antinociceptive activity.

#### Formalin test

**Fig. 2:**
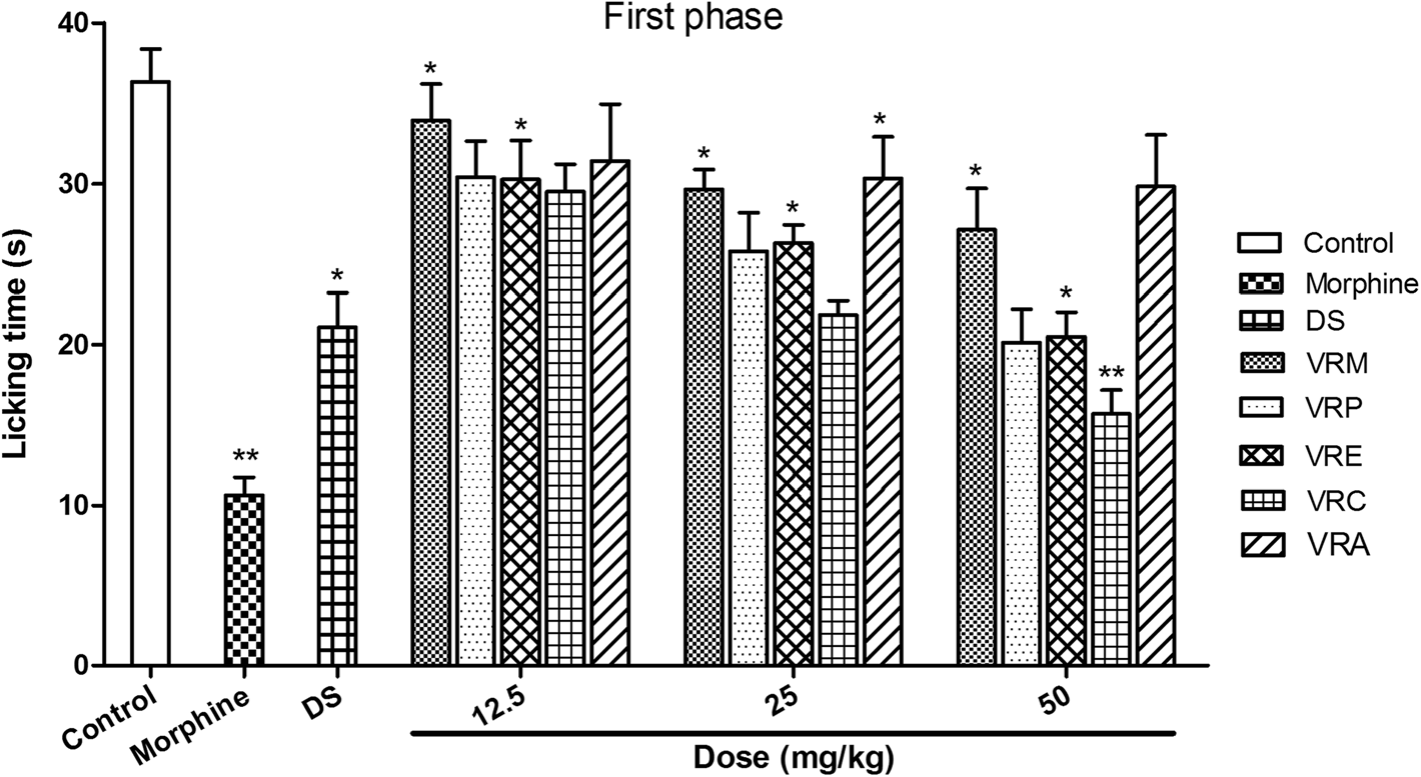
Effect of methanol extract of the *V. roxburghii* root and its different fractions, DS, and morphine (10 mg/kg) on formalin test (first phase). Values are mean ± S.E.M. *p < 0.05 and **p < 0.01, significantly different from control; ANOVA followed Dunnett’s test (n = 6, per group). VRM: *V. roxburghii* methanol extract; VRP: *V. roxburghii* petroleum ether fraction; VRE: *V. roxburghii* ethyl acetate fraction; VRC: *V. roxburghii* chloroform fraction; VRA: *V. roxburghii* aqueous fraction; DS: diclofenac sodium

**Fig. 3:**
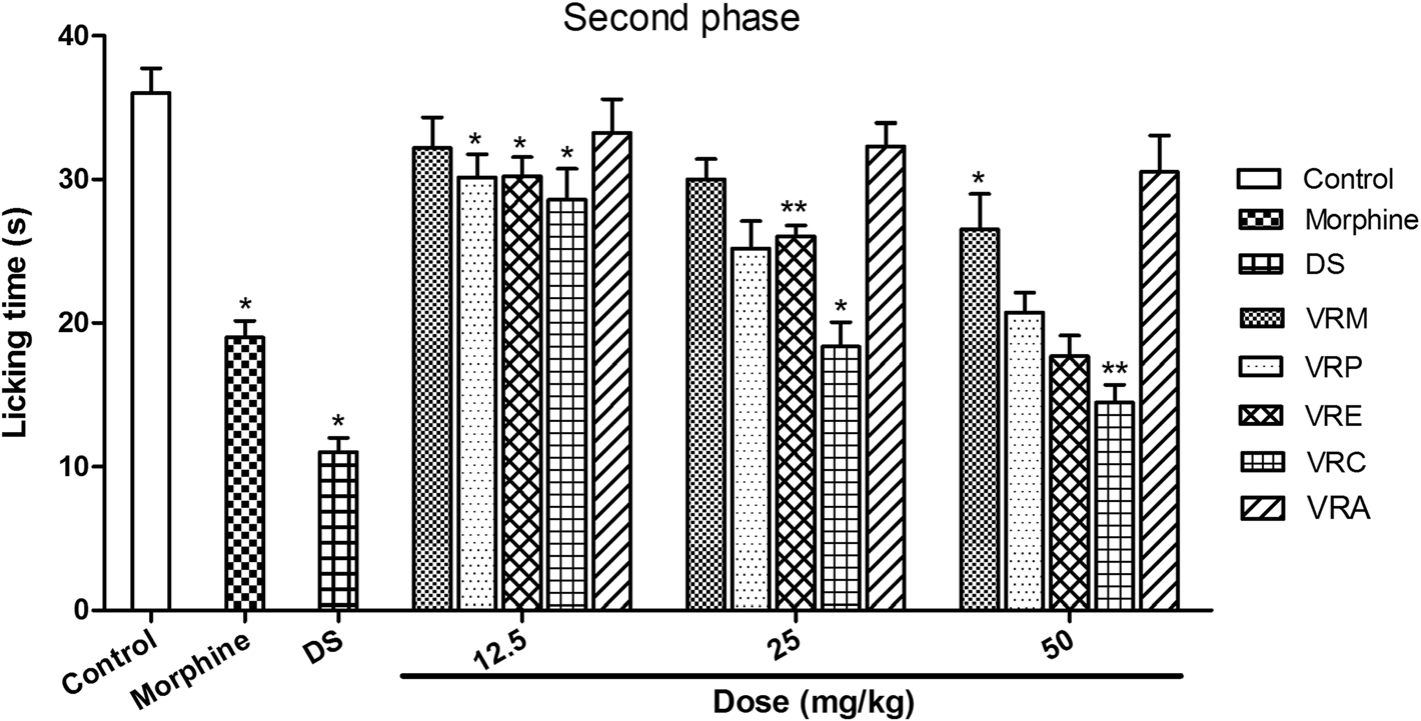
Effect of methanol extract of the *V. roxburghii* root and its different fractions, DS, and morphine (10 mg/kg) on formalin test (second phase). Values are mean ± S.E.M. *p < 0.05 and **p < 0.01, significantly different from control; ANOVA followed Dunnett’s test (n = 6, per group). VRM: *V. roxburghii* methanol extract; VRP: *V. roxburghii* petroleum ether fraction; VRE: *V. roxburghii* ethyl acetate fraction; VRC: *V. roxburghii* chloroform fraction; VRA: *V. roxburghii* aqueous fraction; DS: diclofenac sodium

The biphasic formalin model displays the site and mechanism of action of analgesics represented by neurogenic and inflammatory pain respectively. All the test extract significantly reduced the paw licking time in both phases in a concentration-dependent manner but the reduction was most significant in late phase. The percentage of paw licking time inhibition of VRC in both phases indicated that it possessed potential inhibitory activity. This extract decreased paw licking time by 56.44 and 59.81 % at 50 mg/kg in the first and second phase respectively. The reference drug diclofenac sodium was only effective in the second phase with 70.29 % inhibition at the dose of 10 mg/kg. Morphine reduced the licking time 47.33 and 70.74 % during early and late phases respectively. VRP and VRE also provided higher inhibitory activity that reduced paw licking time by 44.59 and 43.57 % in the early phase comparing with morphine (Fig. 2), as well as 42.44 and 50.94 % in the late phase at 50 mg/kg in comparison with diclofenac sodium (Fig. 3), respectively. VRM and VRA were two test samples amongst five contributed lower activity 6.57 and 13.54 % in first phase while 10.55 and 7.61 % in second phase, respectively.

#### Hot plate test

**Fig. 4:**
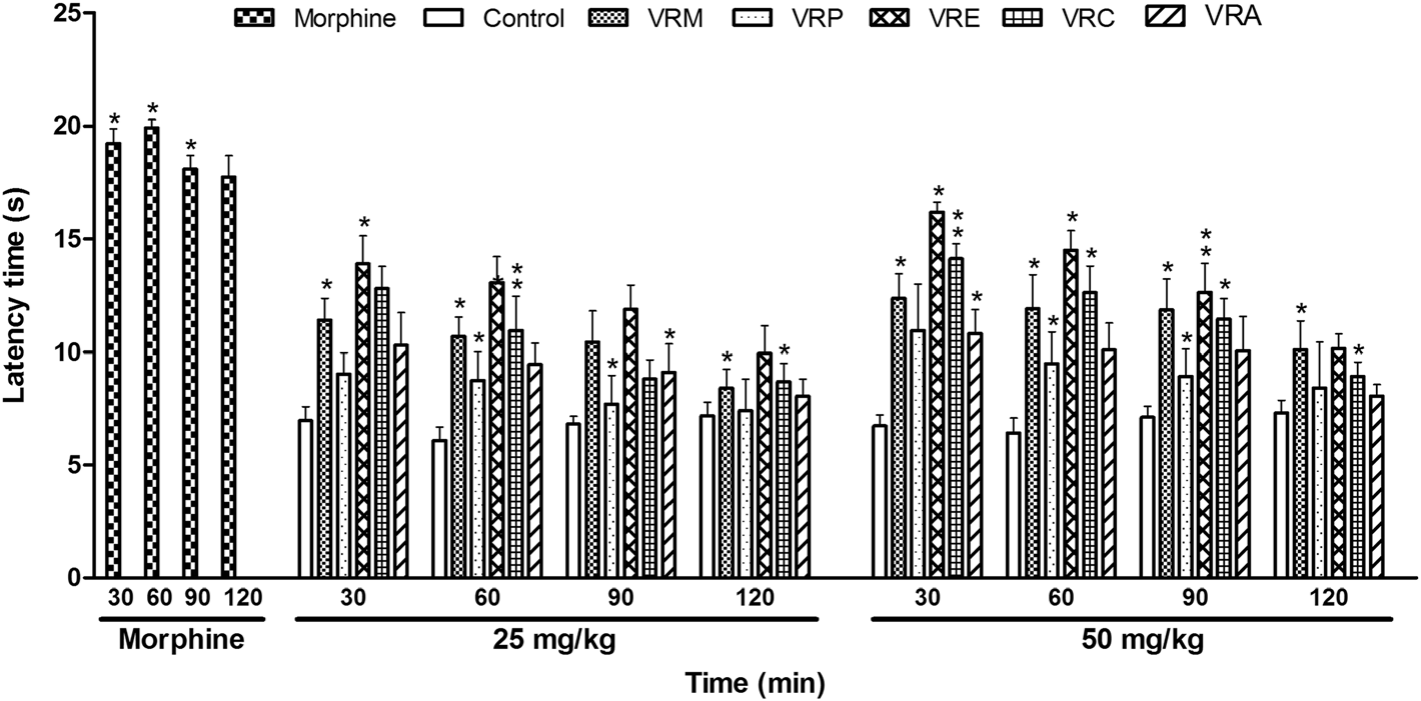
Effect of methanol extract of the *V. roxburghii* root and its different fractions, and morphine on hot plate test. Values are mean ± S.E.M. *p < 0.05 and **p < 0.01, significantly different from control; ANOVA followed Dunnett’s test (n = 6, per group). VRM: *V. roxburghii* methanol extract; VRP: *V. roxburghii* petroleum ether fraction; VRE: *V. roxburghii* ethyl acetate fraction; VRC: *V. roxburghii* chloroform fraction; VRA: *V. roxburghii* aqueous fraction

The result of the hot plate test is shown in Fig. 4. All of the fractions increased the latency time at doses of 25 and 50 mg/kg after 30 min. The effect was dose dependent and among the fractions, ethyl acetate and chloroform extract demonstrated the highest effect (58.43 and 52.34 % respectively) at 50 mg/kg after 30 min. The percentage of inhibition of morphine (10 mg/kg) was significantly higher (p < 0.05) which was 63.77, 69.49, 62.37, and 59.58 % after 30, 60, 90, and 120 min, respectively.

### *In vitro* cytotoxicity assay

**Fig. 5:**
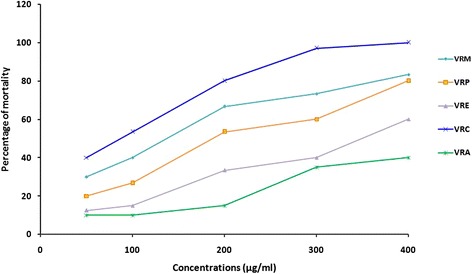
Determination of LC_50_ value of methanol extract of the *V. roxburghii* root and its different fractions from linear correlation between concentrations versus percentage of mortality. VRM: *V. roxburghii* methanol extract; VRP: *V. roxburghii* petroleum ether fraction; VRE: *V. roxburghii* ethyl acetate fraction; VRC: *V. roxburghii* chloroform fraction; VRA: *V. roxburghii* aqueous fraction

The toxicity profile of a compound is a significant indicator for its safe use because of its ability to change the biochemical functions of cells that may finally causes death of cells. Brine shrimps lethality assay therefore, was used to check the cytotoxic effect of all test samples (crude and fractions) in a five different concentrations (50-400 μg/ml) that has been presented in Fig. 5. Results of the lethality test were noted in terms of percentage of mortality of brine shrimp nauplii. Among the fractions, VRC exhibited the lowest LC_50_ value (75.79 μg/ml) while VRA provided the highest LC_50_ value (502.42 μg/ml). The calculated LC_50_ value was 153.72, 223.01, and 343.64 μg/ml for VRM, VRP and VRE respectively. The results reveal that LC_50_ value of all test samples, compared with standard vincristine sulfate (LC_50_ = 0.89 μg/ml) was lower than the cutoff value for cytotoxity.

## Discussion

Nociception is a sensory signal indicating potential harm which most of the time perceived as pain. The sensation of pain develops with the activation of nociceptor mediated by mechanical, thermal or chemical stimuli [25]. Although a number of factors have been identified, enzymes (phospholipase, cyclooxygenase, peroxidase) and stimulation of sensory neurons (spinal and supraspinal) have been implicated as major contributing factors in the generation of pain [26]. In the management of pain, a number of improved analgesic agents have been developed, but there is considerable opportunity regarding innovation of pain reliever.

Our present study is the first time to demonstrate the antinociceptive activity using aerial root of *V. roxburghii* in classical pharmacological models of pain. Although root of *V. roxburghii* is widely used in the folk medicine in all over Bangladesh but the antinociceptive activity of this plant root has not been reported yet. The study of plant species that are traditionally used for the relief of the pain should still be seen as a logical research strategy to investigate new analgesic drugs [27].

Acetic acid-induced writhing response was the first test to evaluate the antinociceptive activity of the root of *V. roxburghii*, is a well recommended protocol in evaluating medicinal agents for their peripherally acting analgesic property. The intraperitoneal administration of acetic acid causes abdominal contractions, whole body movements and twisting of the dorso-abdominal muscles. In this model pain is generated indirectly via proinflammatory agent capsaicin and, endogenous mediators like bradykinin and serotonin which stimulate peripheral nociceptive neurons that are sensitive to NSAIDs and narcotics [28]. The pain stimulus also leads to the release of free arachidonic acid from the tissue phospholipid [29] which is thought to be mediated by peritoneal mast cells [30], acid sensing ion channels [31] and the prostaglandin pathways [32]. The Pain sensation in acetic acid induced writhing response is demonstrated by triggering localized inflammatory response due to release of free arachidonic acid from tissue phospholipids via cyclo-oxygenase (COX), and prostaglandin specifically PGE2 and PGF2 biosynthesis, the level of lipoxygenase products may also increases in peritoneal fluids [33, 34]. These prostaglandin and lipoxygenase products cause inflammation and pain. Inhibition of writhing indicates analgesic effects of a substance mediated by inhibition of prostaglandin synthesis, a peripheral mechanism of pain inhibition [35]. Furthermore, different flavonoids also act as antinociceptive and anti-inflammatory agents due to their ability to inhibit arachidonic acid metabolism [36, 37]. Preliminary phytochemical screening of *V. roxburghii* qualitatively identified the presence of flavonoids, tannins, alkaloids, saponins, phenolics, steroids, and glycosides. Therefore, flavonoids of the extracts might be responsible for antinociceptive activity. The results of our extracts in acetic acid-induced abdominal contraction exhibited prominent inhibition of writhing response. These findings imply deep insights regarding strong peripheral analgesic activity of petroleum ether and ethyl acetate extractives, and their mechanisms of action may be mediated by inhibition of cyclooxygenase activity or prostaglandin synthesis.

Formalin test is one of the appropriate methods to distinguish between the central and peripheral antinociceptive action. In this test, behavioral observations are converted to numerical values [38]. This biphasic model is represented by initial phase, neurogenic (1-5 min) and late phase, inflammatory pain (15-30 min) respectively [39]. In this experiment, different fractions of root of *V. roxburghii* decreased the licking time in both phases, but the effect was more significant in the second phase. Among the five extracts, the most promising effect was observed in case of chloroform and ethyl acetate fractions followed by petroleum ether fraction exerted a significant decrease of licking time in both phases. The suppression of neurogenic and inflammatory pains by the extracts might imply that they contain active analgesic principle that may act as both centrally and peripherally. These findings strongly recommend that the root of this epiphytic plant can be used to manage acute as well as chronic pain. Opioid analgesics exert its antinociceptive effects for both phases where the first phase is more sensitive while NSAIDs seem to suppress only the second phase. A decrease in licking time in both phases therefore, denotes a possible interaction with neurogenic and inflammatory pain modulators.

Hot plate test, thermal nociception model, was used to evaluate central analgesic activity. All of the extracts displayed analgesic effect in the hot plate test but effect of ethyl acetate extract was the highest. As the hot plate test is a specific central antinociceptive test, it is possible that root extracts exert an analgesic effect at least in part through central mechanisms.

Non-prescription use of medicinal plants is reported today as an important health problem, particularly in nephrotoxicity [40]. In this regards, brine shrimps cytotoxicity assay has been considered as a pre-screening assay, and suggested to be a convenient probe for the pharmacological activities of plant extracts [41]. On the basis of correlation between the LC_50_ of the brine shrimp lethality assay and the acute oral toxicity test in mice, Logarto reported that brine shrimp lethality LC_50_ < 10 μg/ml (LD_50_ between 100 and 1000 mg/kg) is considered as the cutoff value of cytotoxicity [42]. In the brine shrimp lethality assay, lower cytotoxicities were found for all extracts compared with the standard drug vincristine sulfate.

## Conclusions

Our findings indicate that root of *V. roxburghii* possess antinociceptive properties with lower toxicity. Therefore, it has been suggested that the compounds, either plant or plant-derived molecules would be an effective candidates for potential drugs that restrict the development of pain. Furthermore, extensive studies are needed to elucidate the exact component and precise mechanisms responsible for these effects.
